# SPIN-Based Linear Temporal Logic Path Planning for Ground Vehicle Missions with Motion Constraints on Digital Elevation Models

**DOI:** 10.3390/s24165166

**Published:** 2024-08-10

**Authors:** Manuel Toscano-Moreno, Anthony Mandow, María Alcázar Martínez, Alfonso José García-Cerezo

**Affiliations:** Institute for Mechatronics Engineering and Cyber-Physical Systems, Robotics and Mechatronics Group, Universidad de Málaga, 29071 Málaga, Spain; mamartinezs@uma.es (M.A.M.); ajgarcia@uma.es (A.J.G.-C.)

**Keywords:** linear temporal logic, SPIN, uneven terrain, uncrewed ground vehicle, digital elevation model, path planning, mission specification

## Abstract

Linear temporal logic (LTL) formalism can ensure the correctness of mobile robot planning through concise, readable, and verifiable mission specifications. For uneven terrain, planning must consider motion constraints related to asymmetric slope traversability and maneuverability. However, even though model checker tools like the open-source Simple Promela Interpreter (SPIN) include search optimization techniques to address the state explosion problem, defining a global LTL property that encompasses both mission specifications and motion constraints on digital elevation models (DEMs) can lead to complex models and high computation times. In this article, we propose a system model that incorporates a set of uncrewed ground vehicle (UGV) motion constraints, allowing these constraints to be omitted from LTL model checking. This model is used in the LTL synthesizer for path planning, where an LTL property describes only the mission specification. Furthermore, we present a specific parameterization for path planning synthesis using a SPIN. We also offer two SPIN-efficient general LTL formulas for representative UGV missions to reach a DEM partition set, with a specified or unspecified order, respectively. Validation experiments performed on synthetic and real-world DEMs demonstrate the feasibility of the framework for complex mission specifications on DEMs, achieving a significant reduction in computation cost compared to a baseline approach that includes a global LTL property, even when applying appropriate search optimization techniques on both path planners.

## 1. Introduction

Formally guaranteeing correctness [1] is crucial for synthesizing effective controllers for complex time-critical ground vehicle missions in applications such as disaster, agriculture, and planetary robotics. Moreover, manageable mission specifications for mobile robots must be concise, human readable, and checkable [2], which requires coping with the incompleteness, ambiguity, and inconsistency of informal human descriptions [3]. Linear temporal logic (LTL) offers a modal formalism that allows describing system properties over linear time using propositional and temporal logical operators [4] with a close relation to natural language, and thus to human reasoning [5]. Furthermore, a system whose properties are expressed by LTL formulas can be formally verified by LTL model checking [3].

Previous works have exploited these qualities of LTL for synthesizing controllers for robotic missions. For instance, [6] developed a framework for controlling linear systems using LTL specifications, extending the LTL controller synthesis from the discrete to continuous domain. In another study, ref. [7] proposed an LTL formula for specifying user-defined high-level behaviors for robots that can react to dynamic environment information. Additionally, ref. [8] extended LTL to simulate collaborative multi-robot environments, emphasizing localization and coordination among robots. Other works have addressed uncertain environments by synthesizing reactive controllers that update obstacle information from sensors during navigation. Thus, ref. [9] developed reactive controllers to manage unpredictable conditions to improve adaptability. Moreover, ref. [10] focused on dynamic updates for synthesized controllers, enhancing system robustness in uncertain environments. In general, these control approaches benefit from LTL specifications, which ensure that the desired task is accomplished if it is feasible.

These properties of LTL have been leveraged in other works to incorporate constraints for deliberative path planning. Thus, ref. [11] studied motion coordination for data gathered through communication and buffer constraints where the high level mission specification for the multi-robot team is performed in LTL. In this sense, ref. [12] analyzed inter-task dependencies, which are crucial for the efficiency of multi-robot systems in coordinated tasks. Recent works have addressed different aspects of environment complexity. In [13], two-dimensional (2D) cells were categorized into five levels of terrain cost for a path planning method for a planetary rover, considering factors such as remaining charge, obstacles, illumination, and communication conditions. Moreover, the T* algorithm [14] is an LTL path planner that uses A* opportunistically to generate an optimal trajectory satisfying a temporal logic formula in a 2D environment. In [15], LTL is used to specify mission requirements for sampling-based path planning using a multi-layered framework that can be suitable for complex environments. In [16], a high-level task planner for bipedal robots used LTL to perform reactive game synthesis between the robot and a dynamic environment with stairs leading to a higher platform. Nevertheless, these works primarily used 2D representations of the environment, so uneven terrain constraints were not explicitly considered. To the best of our knowledge, no previous work has addressed LTL path planning for high-level robot missions on digital elevation models (DEMs).

In general, a single generalized LTL formula for synthesizing a robot controller can integrate both the desired robot behavior and the assumptions about the environment [7,8,10]. However, the implementation of this powerful LTL expressiveness with software tools such as a Simple Promela Interpreter (SPIN) [17] or the Temporal Logic Planning (TuLiP) toolbox [18] can suffer from the state-space explosion problem [17]. The work in [19] addressed scalability for task allocation with multiple heterogeneous robots from a global LTL specification, but individual path planning is performed at a lower level. Furthermore, ref. [20] reduced the computation time of the path planning by modeling robot mobility as an abstract weighted transition system.

In this article, we propose a SPIN-based LTL path planning approach for uncrewed ground vehicle (UGV) missions on uneven terrain by defining a system model that includes motion constraints and performing model checking of the mission specification’s LTL property. Since UGV motion constraints for the DEM are incorporated into the system model, they can be omitted from LTL model checking for path planning according to the mission specification. Moreover, we define two SPIN-efficient general LTL formulas for representative UGV missions to reach goals in a specified order or an unspecified order, respectively. The proposed planner is implemented using the open-source model checker SPIN with customized search optimization parameters. Validation experiments conducted on synthetic and real-world DEMs demonstrate the feasibility of the LTL path planning framework for complex mission specifications, achieving a significant reduction in computation cost compared to a SPIN-based baseline approach that considers a global LTL property including both motion constraints and mission specification.

The main contributions of this work are as follows:

- The definition of a system model as a transition system that includes UGV asymmetric slope traversability and maneuverability on uneven terrains represented as DEMs.
- A SPIN-based LTL path planner for UGV missions with customized parameterization for effective search optimization. This LTL planner uses the system model and an LTL property that defines only the UGV mission specification based on DEM goals.
- Two general LTL formulas for specifying two types of UGV missions to reach a DEM partition set, either in a specified order or an unspecified order. These formulas use the *until* operator, which enables efficient SPIN state-space exploration.

The remainder of the article is organized as follows. Section 2 reviews LTL concepts for path planning, fulfilling mission specifications. Section 3 proposes the design of the LTL-based path planner for UGV missions on uneven DEMSs. Section 4 describes the experimental methodology. Section 5 discusses the experimental results. Finally, Section 6 offers conclusions and ideas for future work.

## 2. Review of LTL Concepts

This section reviews fundamental concepts of LTL, model checking, and LTL path planning for high-level UGV missions.

### 2.1. Linear Temporal Logic

Linear temporal logic (LTL) is a logical formalism that expresses the behavior of a reactive system over linear time, i.e., the relative order of events. The basic elements of LTL are a set of atomic propositions Π, and Boolean and temporal operators. An atomic proposition π∈Π is a statement or assertion that must be true or false.

A well-formed LTL formula ϕ is a finite sequence of symbols satisfying the following grammar:

(1) $$\phi ::=\;\;\pi \;|\;\neg \phi \;|\;\phi_{1}\;\;\;\vee \;\;\;\phi_{2}\;\left|\;\;\;◯\;\;\phi \;\right|\;\phi_{1}\;U\;\;\;\phi_{2}\;,$$

where ¬ and ∨ are Boolean operators for *negation* and *disjunction*, respectively, and ◯ and U are the temporal operators *next* and *until*, respectively. This grammar allows defining other LTL derived operators such as the following:

(2) $$\begin{array}{ccc} ⊤\;(\mathrm{or}\;\mathrm{true}) & \overset{\mathrm{def}}{=} & \phi \;\;\;\vee \;\;\;\;\;\neg \;\;\phi \;,\\ ⊥\;(\mathrm{or}\;\mathrm{false}) & \overset{\mathrm{def}}{=} & \;\;\neg \;\;⊤\;,\\ \phi_{1}\;\;\;\wedge \;\;\;\phi_{2} & \overset{\mathrm{def}}{=} & \;\;\neg \;\;(\;\;\neg \;\;\phi_{1}\;\;\;\vee \;\;\;\;\;\neg \;\;\phi_{2})\;,\\ ◊\phi & \overset{\mathrm{def}}{=} & ⊤\;U\;\;\;\phi \;,\\ □\phi & \overset{\mathrm{def}}{=} & \;\;\neg \;\;◊\;\;\neg \;\;\phi \;, \end{array}$$

where ∧ denotes *conjunction*, and ◊ and −□ are the temporal operators *eventually* and *always*, respectively. Unitary operators have the highest precedence, and U takes precedence over other binary operators and is left-associative, e.g., $\phi_{1}\;U\;\;\;\phi_{2}\;U\;\;\;\phi_{3}$ stands for $\left(\phi_{1}\;U\;\;\;\phi_{2}\right)\;U\;\;\;\phi_{3}$.

The semantics of ϕ are defined over infinite traces. A trace σ is a sequence $\sigma =\sigma_{1}\sigma_{2}\cdots$ of truth assignments to Π, where $\sigma_{i}\subset \Pi$. A suffix trace is defined as $\sigma \left(i\right)=\sigma_{i}\sigma_{i+1}\cdots$; thus, σ=σ(1). For a given trace σ, the notation σ⊧ϕ denotes that σ satisfies ϕ. The satisfaction relationship ⊧ between σ(i) and ϕ is defined as follows:

(3) $$\begin{array}{cc} \sigma (i)⊧\pi & \mathrm{iff}\;\pi \in \sigma {\left(i\right)}_{1}\;,\\ \sigma (i)⊧\;\;\neg \;\;\phi & \mathrm{iff}\;\sigma (i)\;/\;⊧\phi \;,\\ \sigma \left(i\right)⊧\phi_{1}\;\;\;\vee \;\;\;\phi_{2} & \mathrm{iff}\;\sigma \left(i\right)⊧\phi_{1}\;\mathrm{or}\;\sigma \left(i\right)⊧\phi_{2}\;,\\ \sigma (i)⊧\;\;◯\;\;\phi & \mathrm{iff}\;\sigma (i+1)⊧\phi \;,\\ \sigma \left(i\right)⊧\phi_{1}\;U\;\;\;\phi_{2} & \mathrm{iff}\;\exists k>i,\sigma \left(k\right)⊧\phi_{2}\;\mathrm{and}\;\\ \; & \;\forall \left(i\leq j<k\right),\sigma \left(j\right)⊧\phi_{1}\;. \end{array}$$

### 2.2. LTL Model Checking

Formal model checking systematically checks whether a formal specification property ϕ is satisfied for a system model $A_{S}$ defined as a transition system without terminal states. In LTL model checking, the states of this system are defined from Π, and ϕ as a deterministic finite automaton $A_{\;\;\neg \;\;\phi }$ with accepting states that recognizes the language $L(A_{\;\;\neg \;\;\phi })$ formed by the set of non-admissible traces. Since LTL semantics are defined over traces, model checking is reduced to a reachability problem in the product automaton $A_{S}⊗A_{\;\;\neg \;\;\phi }$. Then,

(4) $$A_{S}⊧\phi \;\mathrm{iff}\;\mathrm{finite}\_\mathrm{traces}\left(A_{S}\right)\;\cap \;L\left(A_{\;\;\neg \;\;\phi }\right)=⌀\;,$$

i.e., the correctness of $A_{S}$ is verified with respect to ϕ if and only if there is no intersection between the finite traces of $A_{S}$ and $L(A_{\;\;\neg \;\;\phi })$.

Model checker tools, such as the Uppsala-Aalborg (Uppaal) suite [21], the New Symbolic Model Verifier (NuSMV) [22], TuLiP [18], or SPIN [23], automatically determine whether the model truly satisfies a given property. When the property is not fulfilled, they provide a counterexample trace, i.e, an $A_{S}$ execution path that reaches an undesired state.

Even if model checking has a sound mathematical foundation based on graph theory, data structures, and logic, model checkers suffer from the state-space explosion problem when the number of states needed to accurately model realistic systems or complex properties exceeds available computer memory [17].

SPIN offers features to cope with the state-space explosion and, starting from version 6.5.0, allows the conversion of LTL formulas of arbitrary length. In particular, SPIN includes search optimization techniques [17,24] to reduce the following: (i) The number of explored states; (ii) The memory required. To reduce the number of explored states, SPIN introduces partial order reduction and statement merging (atomic and deterministic step sequences). For memory reduction, SPIN includes the following state vector compression methods:

- Lossless (collapse compression and minimized finite state recognizer), which preserve exhaustive verification capability at the cost of increased runtime requirements.
- Lossy (bitstate hashing and hash-compact methods) to further reduce memory requirements. However, this approach does not guarantee exhaustive verification, which may result in a suboptimal solution.

SPIN supports Promela (acronym for Process Meta Language), a high-level language for modeling system and LTL properties. Promela is a C-like meta-language with very limited expressiveness in order for verification to be efficient, and supports embedded C-code as part of the model specification.

### 2.3. LTL Path Planning for High-Level UGV Missions

Path planning for high-level UGV missions determines a motion sequence that a UGV must aim to fulfill a given mission specification. This specification can be formulated as an LTL property ϕ, describing the order or frequency (*eventually* or *infinitely often*) in which truth assignments to atomic propositions in Π must occur for a correct mission execution.

LTL path planning for high-level UGV missions is a decision-making process through a nondeterministic $A_{S}$ using a model checker for ¬ϕ [25]. The nondeterministic transitions of $A_{S}$ correspond to UGV motions limited by the neighborhood (typically, four neighbors) regardless of the mission. The model checker searches for a counterexample trace σ⊧ϕ that, by not satisfying ¬ϕ, fulfills the given LTL property ϕ. Thus, the transitions that produce σ define the UGV motion sequence that satisfies the specified mission.

## 3. DEM-Based LTL Path Planning for High-Level UGV Missions

This section presents the core methodology of our framework, detailing the design of the LTL-based path planner for UGV missions on uneven terrains. First, we introduce the definition of the system model according to motion constraints. Next, the SPIN-efficient general LTL formulas designed for high-level UGV missions are proposed. Then, we describe the workflow of LTL model checking using the SPIN tool, emphasizing the optimization techniques employed to cope with state-space explosion. Finally, the section concludes with the interpretation of the LTL model checking results for path planning for UGV mission on DEMs.

### 3.1. System Model According to Motion Constraints

In this paper, we address path planning for UGV missions on uneven terrains that can be modeled as a DEM, which can be represented by a matrix $Z\in M_{n\times m}\left(R\right)$ with the altitude in a map projection system (regular grid with distance δ between points, i.e., DEM cell resolution). We propose a path planner for high-level UGV mission specifications based on DEM and LTL model checking, where UGV motion between DEM cells is constrained by asymmetric slope traversability conditions [26], which depends on both the robot and the terrain [27,28]. Traversability is defined as matrix $T\in M_{n\times m}\left({\left\{0,1\right\}}^{\ell }\right)$, where each element t(i,j) is a Boolean *ℓ*-vector representing motion feasibility from (i,j) to its *ℓ*-neighbors according to slope α, computed as

(5) $$\alpha =\arctan\left(\frac{z\left(i_{o},j_{o}\right)-z\left(i,j\right)}{\delta \sqrt{{\left(i_{o}-i\right)}^{2}+{\left(j_{o}-j\right)}^{2}}}\right)\;.$$

Elements of t(i,j) are denoted by $t(i,j,\theta_{o})$, ∀o∈{1,2,⋯,ℓ}, where $\theta_{o}$ is the outward heading toward neighboring cells $\left(i_{o},j_{o}\right)$, i.e., a multiple of π/2 or π/4 rad for ℓ=4 or ℓ=8, respectively:

(6) $$\begin{array}{ccc} t(i,j,\theta_{o})=⊤ & \mathrm{iff}\; & \alpha_{\min}\leq \alpha \leq \alpha_{\max}\;, \end{array}$$

and $\alpha_{\min}$ and $\alpha_{\max}$ are the minimum and maximum UGV slope constraints, respectively. We adopted null heading for the eastern direction and counterclockwise angle increments.

**Figure 1:**
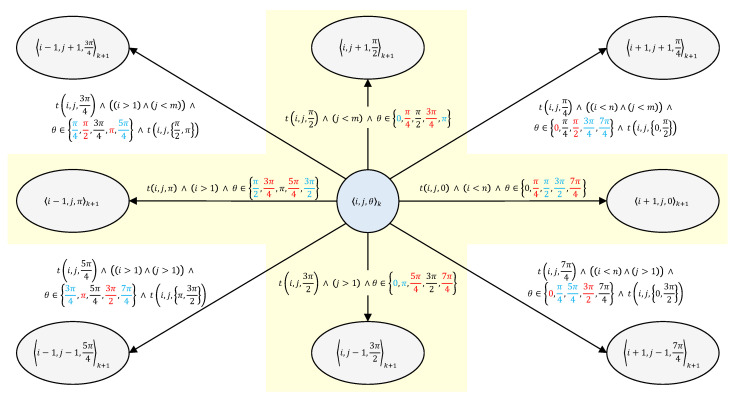
Representation of $A_{S}$ transitions from *k* to k+1. For ℓ=4, only the shadowed yellow area is considered.

The system representing the UGV motion on a DEM is modeled as $A_{S}$, a nondeterministic transition system whose states at discrete time *k* are given by ${\langle i,j,\theta \rangle }_{k}$, where (i,j) represents cell coordinates and θ is the inward heading. As for transitions, we adopted reducing the number of valid transitions by defining motion constraints in order to cope with the state-space explosion and to reduce the computational cost. In particular, as shown in Figure 1, transitions in $A_{S}$ are defined by a conjunction of four Boolean constraints: (a) slope traversability *T*; (b) DEM n×m limits; (c) the set Θ of allowed on-cell turns $\left(\theta_{o}-\theta \right)$, which is Θ={0,±π/2} for ℓ=4 (i.e., removing red values from Figure 1), and can be either Θ={0,±π/4} (i.e., removing blue values from Figure 1) or Θ={0,±π/4,±π/2} for ℓ=8, depending on UGV kinematics constraints; and (d) a specific constraint for diagonal transitions requiring that the adjacent four neighboring cells are traversable (e.g., the transition to $\theta_{k+1}=\pi /4$ also depends on traversabilities to $\theta_{k+1}=0$ and $\theta_{k+1}=\pi /2$).

### 3.2. LTL Formulas for High-Level UGV Mission Specifications

In this paper, we introduce two SPIN-efficient general LTL formulas for representative high-level UGV missions. With this purpose, we define a set X of non-overlapping state partitions. Each partition $X_{p}=\left\{\langle i_{p1},j_{p1},\theta_{p1}\rangle ,\langle i_{p2},j_{p2},\theta_{p2}\rangle ,\cdots \right\}\in X$ consists of a set of states to which an atomic proposition $\pi_{p}$ is associated:

(7) $$\begin{array}{ccc} \pi_{p}=⊤ & \mathrm{iff} & {\langle i,j,\theta \rangle }_{k}\in X_{p}\;. \end{array}$$

Only mission-relevant DEM cells need to be considered in the partitions. The mission specification for a partition is fulfilled if the UGV can reach any state within the partition with the corresponding inward heading.

Regarding the optimization criterion, SPIN imposes minimization of the number of counterexample states. Thus, the objective of the path planner for high-level UGV mission is to obtain a path with the minimum number of $A_{S}$ transitions that reaches all goal partitions of a specified set $G=\{G_{1},G_{2},\ldots ,G_{\left|G\right|}\}\subseteq X$. Furthermore, mission specifications can include (1) a partition of forbidden states, F∈X:F∉G associated to atomic proposition $\pi_{f}$,

(8) $$\begin{array}{ccc} \pi_{f}=⊤ & \mathrm{iff} & {\langle i,j,\theta \rangle }_{k}\in F\;, \end{array}$$

and/or (2) the requirement to return to the initial cell coordinates $\left(i_{0},j_{0}\right)$ after the mission is completed using an atomic proposition $\pi_{r}$,

(9) $$\begin{array}{ccc} \pi_{r}=⊤ & \mathrm{iff} & \left(\left(i_{k},j_{k}\right)=\left(i_{0},j_{0}\right)\right)\;\;\;\wedge \;\;\;\left(\theta_{k}\in \Theta_{r}\right)\;, \end{array}$$

where $\Theta_{r}$ is the specified set of allowed inward headings for returning.

When verifying complex systems with SPIN, it is crucial to choose LTL formulas that enable efficient state-space exploration [25]. LTL formulas that use the temporal operator U (*until*) explicitly enforce a strict event order and avoid certain conditions, which reduces the state space, making it easier for SPIN to find counterexamples. Conversely, LTL formulas with global restrictions and nested eventualities (e.g., $□\neg \pi_{f}\wedge ◊\left(\pi_{1}\wedge ◊\left(\pi_{2}\wedge ◊\left(\pi_{3}\wedge ◊\pi_{4}\right)\right)\right)$) can slow down the state-space exploration process. Therefore, we propose two general LTL formulas using U for reaching goal partitions G:

(1) In a given order (i.e., $G_{1}$ then $G_{2}$, and so on),
  (10) $$\;\phi_{g1}\;\overset{\mathrm{def}}{=}\;\;\;\;\neg \;\;\pi_{f}\;\;U\;\;\;\;\pi_{r}\;\;U\;\;\;\overset{1}{\underset{ı=|G|}{⋃}}\pi_{ı}\;,$$
  where $\overset{1}{\underset{ı=|G|}{⋃}}\pi_{ı}=\pi_{\left|G\right|}\;\;U\;\;\;\;\pi_{|G|-1}\;\;U\;\;\;\cdots \;U\;\;\;\;\pi_{1}$
(2) In an unspecified order,
  (11) $$\;\begin{array}{cc} \phi_{g2} & \overset{\mathrm{def}}{=}\;\;\overset{\left|G\right|}{\underset{ı=1}{⋀}}\left(\;\;\;\neg \;\;\pi_{f}\;\;U\;\;\;\;\pi_{r}\;\;U\;\;\;\;\pi_{ı}\;\right)\;\\ \; & =\left(\;\;\;\neg \;\;\pi_{f}\;\;U\;\;\;\;\pi_{r}\;\;U\;\;\;\;\pi_{1}\;\right)\;\;\;\wedge \;\;\;\left(\;\;\;\neg \;\;\pi_{f}\;\;U\;\;\;\;\pi_{r}\;\;U\;\;\;\;\pi_{2}\;\right)\;\\ \; & \;\;\;\;\wedge \;\;\;\cdots \;\;\;\wedge \;\;\;(\;\;\;\neg \;\;\pi_{f}\;\;U\;\;\;\;\pi_{r}\;\;U\;\;\;\;\pi_{\left|G\right|}\;)\;. \end{array}$$
   

Moreover, the following three simplifying conditions could be considered in actual missions:

- There is a single goal partition, i.e., |G|=1;
- There is no forbidden partition (F=⌀), i.e., $\pi_{f}=⊥$;
- The UGV must not return to the initial cell, i.e., $\pi_{r}=⊤$;

Using different combinations of these simplifying conditions in Equations (10) and (11) results in the well-known simple LTL formulas presented in Table 1. This result corroborates the generality of the proposed formulas, which can be applied for more complex missions.

### 3.3. LTL Model Checking Workflow with SPIN

**Figure 2:**
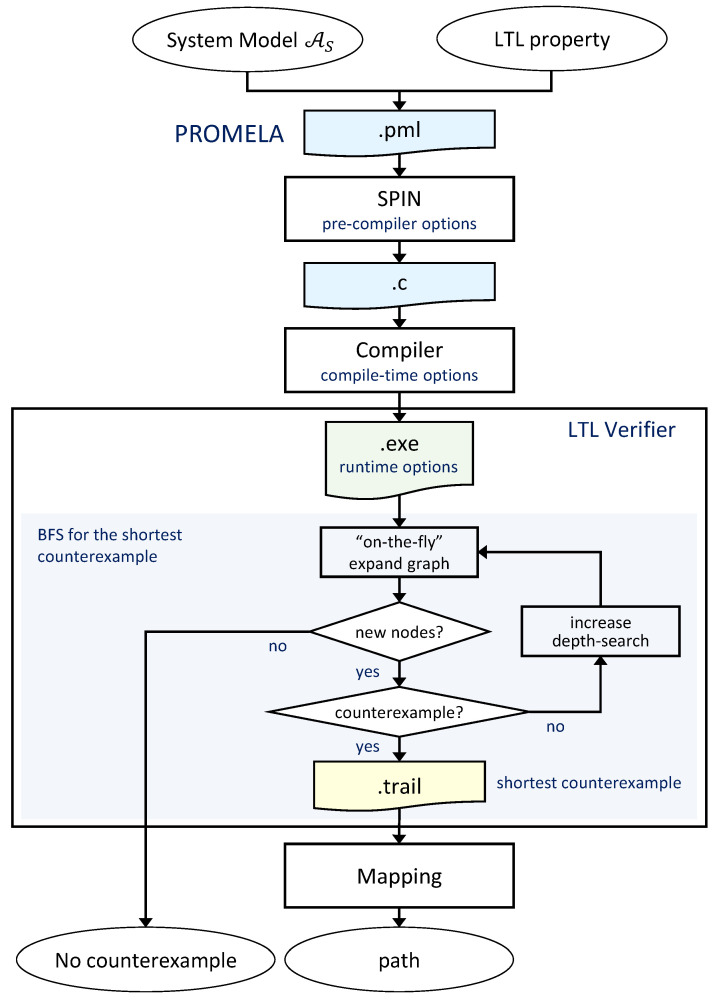
Simple Promela Intepreter (SPIN)-based LTL model checking workflow with breadth-first search for the shortest trace σ⊧ϕ.

In this work, we adopted the SPIN workflow with breadth-first search (BFS) [17] for synthesizing an LTL path planner for high-level UGV missions (see Figure 2). Unlike the default depth-first search, which is more suitable for model checking than for planning, BFS can find a counterexample with the shortest trace at a lower computational cost.

This workflow begins with using Promela to model $A_{S}$ and the LTL property to be verified. This Promela source file is used by SPIN to generate an LTL verification program in ANSI C. Then, our compile time options for specific search optimization techniques are partial order reduction, the BFS algorithm, (-DBFS) and lossless collapse compression (-DCOLLAPSE). Then, an iterative LTL verification process is performed until either no new nodes are added to the unweighted search graph (i.e., no counterexample is found) or a trail file is generated with the shortest counterexample (i.e., $A_{S}$ does not satisfy the LTL property).

### 3.4. LTL Model Checking for Synthesizing a Path Planner for UGV Missions

Since $A_{S}$ was designed considering motion constraints, these can be omitted from LTL model checking for path planning that fulfills the high-level UGV mission. The LTL property used to verify in the LTL model checking workflow (see Figure 2) is a negated LTL mission specification ¬ϕ, i.e., the negation of (10) or (11) (or other simplified or derived formula from these). For LTL path planning, “no counterexample” means that the UGV mission cannot be satisfied; otherwise, the trail file returns the shortest counterexample, which needs to be mapped from the product automaton states to the system model states to obtain a DEM path.

## 4. Experimental Methodology

This section describes the hardware and software used in the experiments, defines a baseline path planner for comparison, and presents mission specifications for a synthetic DEM and a larger-scale real-world DEM.

### 4.1. Hardware and Software

The DEM-based LTL path planner was executed on a 2.10 GHz i7-13700F processor made by Intel Corp. (Santa Clara, CA, USA) with 16 GB of RAM, running Microsoft© Windows 11 Pro. The required software was installed in a container on Docker Desktop v.4.32.0 including Ubuntu 22.04.4 LTS and MATLAB R2023b by Mathworks (Natick, MA, USA) to run the main planner script and generate graphical representations. SPIN v6.5.2 was used as the model checker, gcc 11.4.0 as the C compiler, and Promela to (1) describe the system models with embedded C-code as part of the model specification and (2) formulate DEM-based high-level UGV missions as LTL properties.

### 4.2. Comparison with a Baseline Path Planner

In this article, we propose an approach for SPIN-based LTL path planning on DEMs by defining a system model that incorporates the set of UGV motion constraints, allowing these constraints to be omitted from LTL model checking. This contrasts with previous works in LTL path planning [7,8,10], where a single generalized LTL formula integrates both the desired robot behavior and the environment assumptions.

To the best of our knowledge, no previous works have addressed LTL path planning on DEMs. Therefore, for comparison purposes, we define a baseline SPIN-based LTL path planner with a generalized LTL formula integrating mission specification and motion constraints on DEMs, and a system model that only considers cell neighborhoods for transitions.

In this baseline planner, the generalized LTL formula is

(12) $$\phi_{\mathrm{base}}\;\overset{\mathrm{def}}{=}\;\;\phi \;\;\;\;\wedge \;\;\;\;□\left(\;\pi_{a}\;\;\;\;\wedge \;\;\;\;\pi_{b}\;\;\;\;\wedge \;\;\;\;\pi_{c}\;\;\;\;\wedge \;\;\;\;\pi_{d}\;\right)\;,$$

which means that the mission specification ϕ must be satisfied, and all motion constraints must *always* be fulfilled. In particular, the truth assignments to the four motion constraints described in Section 3.1 are defined as atomic propositions $\pi_{a},\pi_{b},\pi_{c}$, and $\pi_{d}$, respectively, from the current ${\langle i,j,\theta \rangle }_{k}$ and previous ${\langle i,j,\theta \rangle }_{k-1}$ model states, as follows:

(13) $$\begin{array}{ccc} \pi_{a}=⊤ & \mathrm{iff} & \left(k=0\right)\;\;\;\vee \;\;\;\;t\left(i_{k-1},j_{k-1},\theta_{k}\right)\;,\;\\ \pi_{b}=⊤ & \mathrm{iff} & \left(1\leq i_{k}\leq n\right)\;\;\;\wedge \;\;\;\left(1\leq j_{k}\leq m\right)\;,\;\\ \pi_{c}=⊤ & \mathrm{iff} & \left(k=0\right)\;\;\;\vee \;\;\;\left((\theta_{k}-\theta_{k-1})\in \Theta \right)\;,\;\\ \pi_{d}=⊤ & \mathrm{iff} & \left(k=0\right)\;\;\;\vee \;\;\;\left(\theta_{k}\in \left\{0,\pi /2,\pi ,3\pi /2\right\}\right)\\ \; & \; & \;\;\;\vee \;\;\;\left(t\left(i_{k-1},j_{k-1},\theta_{k}\right)\;\;\;\;\wedge \;\;\;\;t\left(i_{k-1},j_{k-1},\theta_{k}\pm \pi /4\right)\right). \end{array}$$

### 4.3. Mission Specifications in a Synthetic DEM

**Figure 3:**
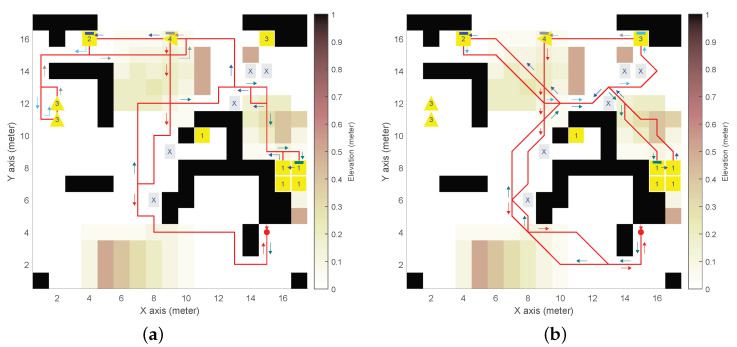
Resulting path for example mission $\phi_{1}$ in synthetic digital elevation model (DEM): (**a**) 4-neighborhood (ℓ=4) and Θ={0,±π/2} rad; (**b**) 8-neighborhood (ℓ=8) and Θ={0,±π/4,±π/2} rad.

**Figure 4:**
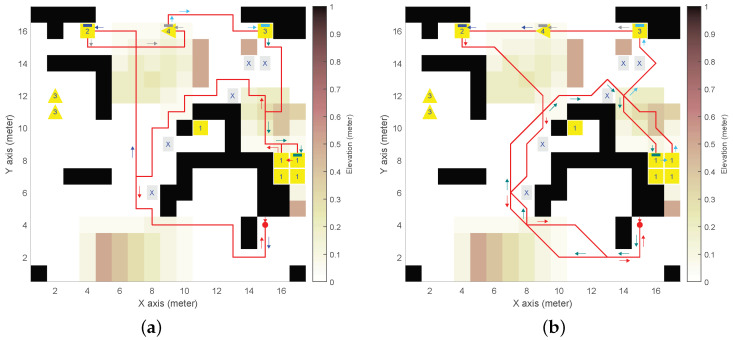
Resulting path for example mission $\phi_{2}$ in synthetic DEM: (**a**) 4-neighborhood (ℓ=4) and Θ={0,±π/2} rad; (**b**) 8-neighborhood (ℓ=8) and Θ={0,±π/4,±π/2} rad.

**Figure 5:**
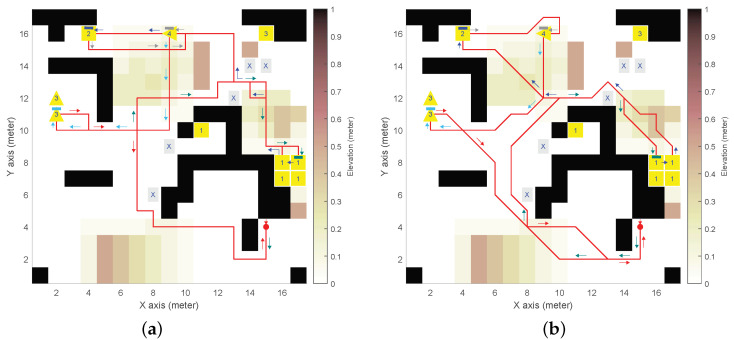
Resulting path for example mission $\phi_{3}$ in synthetic DEM: (**a**) 4-neighborhood (ℓ=4) and Θ={0,±π/2} rad; (**b**) 8-neighborhood (ℓ=8) and Θ={0,±π/4,±π/2} rad.

**Figure 6:**
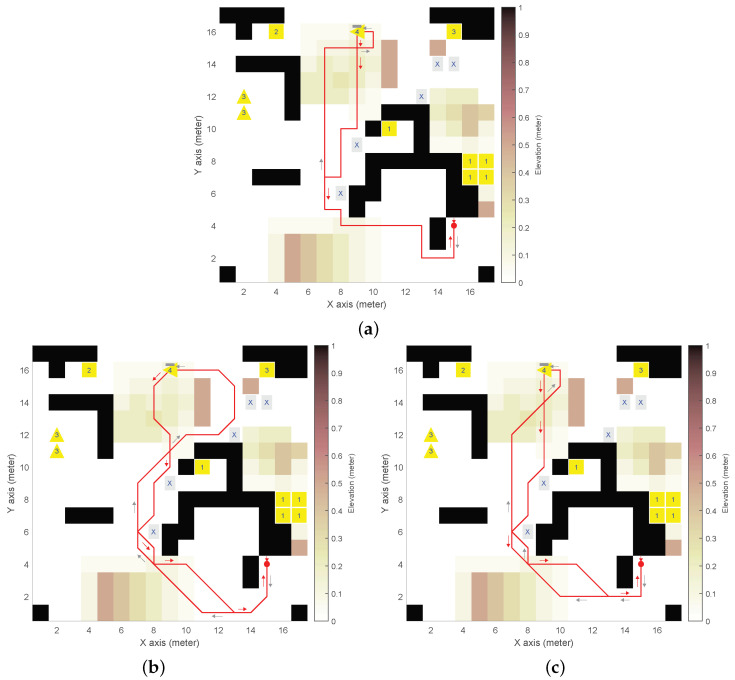
Resulting path for example mission $\phi_{4}$ in synthetic DEM: (**a**) 8-neighborhood (ℓ=4) and Θ={0,±π/2} rad; (**b**) 8-neighborhood (ℓ=8) and Θ={0,±π/4} rad; (**c**) 8-neighborhood (ℓ=8) and Θ={0,±π/4,±π/2} rad.

We defined a synthetic DEM with obstacles, mounds, and slopes (see Figure 3, Figure 4, Figure 5 and Figure 6), with n×m=17×17 and δ= 1 m. The DEM has a forbidden partition F indicated by cells marked with an “X”, and four mission-oriented partitions, $X_{1}$ to $X_{4}$, represented by cells labeled with corresponding numbers.

Partition $X_{1}$ (and also $X_{3}$) exemplifies cells from different regions sharing the same role in the mission (e.g., charging stations or communication relays that could be distributed throughout the environment). Moreover, partitions $X_{3}$ and $X_{4}$ need to be given inward headings (indicated by triangle cell labels).

As for the UGV, the initial state ${\langle i,j,\theta \rangle }_{0}=\langle 15,4,3\pi /2\rangle$ is depicted as a red spot with an arrow for the heading. We adopted UGV slope constraints $\alpha_{\min}=-\pi /18\;\mathrm{rad}\;\left(-10{}^{\circ}\right)$ for downhill and $\alpha_{\max}=\pi /12\;\mathrm{rad}\;\left(15{}^{\circ}\right)$ for uphill, which are representative of asymmetric behaviour of actual UGVs [26].

Four distinctive high-level UGV missions were analyzed:

(1) Example of general LTL formula $\phi_{g1}$, Equation (10), see Figure 3.
  *The UGV must eventually reach $X_{1}$ to $X_{4}$ in the given order, avoiding F, to finally return to $\left(i_{0},j_{0}\right)$ with any inward heading (i.e., $\Theta_{r}=\left\{\theta \;,\;0\leq \theta \leq 2\pi \right\}$)*:
  (14) $$\begin{array}{c} \phi_{1}\;\overset{\mathrm{def}}{=}\;\;\;\neg \;\;\pi_{f}\;\;U\;\;\;\;\pi_{r}\;\;U\;\;\;\;\pi_{4}\;\;U\;\;\;\;\pi_{3}\;\;U\;\;\;\;\pi_{2}\;\;U\;\;\;\;\pi_{1}\\ \;=\left(\left(\left(\left(\;\;\neg \;\;\pi_{f}\;\;U\;\;\;\;\pi_{r}\right)\;\;U\;\;\;\;\pi_{4}\right)\;\;U\;\;\;\;\pi_{3}\right)\;\;U\;\;\;\;\pi_{2}\right)\;\;U\;\;\;\;\pi_{1}\;. \end{array}$$
  To enhance clarity, extra parentheses have been added to the LTL formula, even though the LTL grammar in Section 2.1 specifies that the U operator is left-associative, as is typical in SPIN’s interpretation.
(2) Example of general LTL formula $\phi_{g2}$, Equation (11), see Figure 4.
  *The UGV must eventually reach $X_{1}$ to $X_{4}$ with no specific order, avoiding F, to finally return to $\left(i_{0},j_{0}\right)$ with any inward heading*:
  (15) $$\begin{array}{c} \phi_{2}\;\overset{\mathrm{def}}{=}\;\left(\;\;\neg \;\;\pi_{f}\;\;U\;\;\;\;\pi_{r}\;\;U\;\;\;\;\pi_{1}\;\right)\;\;\;\wedge \;\;\;\left(\;\;\neg \;\;\pi_{f}\;\;U\;\;\;\;\pi_{r}\;\;U\;\;\;\;\pi_{2}\;\right)\\ \;\;\;\;\wedge \;\;\;\left(\;\;\neg \;\;\pi_{f}\;\;U\;\;\;\;\pi_{r}\;\;U\;\;\;\;\pi_{3}\;\right)\;\;\;\wedge \;\;\;\left(\;\;\neg \;\;\pi_{f}\;\;U\;\;\;\;\pi_{r}\;\;U\;\;\;\;\pi_{4}\;\right)\;. \end{array}$$
(3) Example of a combination of Equations (10) and (11), see Figure 5.
  *The UGV must eventually reach $X_{1}$, then $X_{2}$, and then $X_{3}$ and $X_{4}$ with no specific order, avoiding F, to finally return to $\left(i_{0},j_{0}\right)$ with any inward heading*:
  (16) $$\begin{array}{c} \phi_{3}\;\overset{\mathrm{def}}{=}\;\left(\left(\;\;\neg \;\;\pi_{f}\;\;U\;\;\;\;\pi_{r}\;\;U\;\;\;\;\pi_{4}\;\right)\;\;\;\wedge \;\;\;\left(\;\;\neg \;\;\pi_{f}\;\;U\;\;\;\;\pi_{r}\;\;U\;\;\;\;\pi_{3}\;\right)\right)\\ \;\;U\;\;\;\;\pi_{2}\;\;U\;\;\;\;\pi_{1}\;. \end{array}$$
(4) Example of a simplified LTL formula (Table 1), see Figure 6.
  *The UGV must eventually reach $X_{4}$, avoiding F, to finally return to $\left(i_{0},j_{0}\right)$ with any inward heading*:
  (17) $$\phi_{4}\;\overset{\mathrm{def}}{=}\;\;\;\neg \;\;\pi_{f}\;\;U\;\;\;\;\pi_{r}\;\;U\;\;\;\;\pi_{4}\;.$$

### 4.4. Mission Specification in a Real DEM

**Figure 7:**
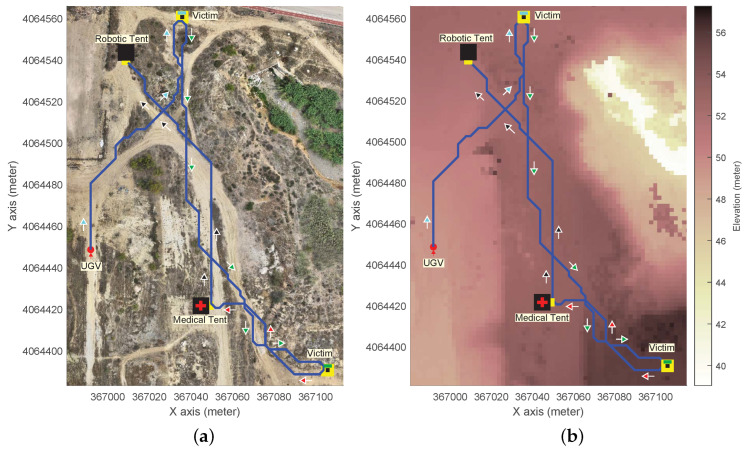
Example of path planning for a search-and-rescue mission specification using formula $\phi_{5}$ in a real-world DEM, with bitstate hashing compression, 8-neighborhood (ℓ=8), and Θ={0,±π/4} rad. (**a**) Aerial orthophoto. (**b**) Digital elevation model.

Additionally, to evaluate planning performance in a larger-scale real-world DEM, we used an n×m=292×232 with δ= 2 m obtained from an aerial ortophoto (see Figure 7). This DEM captures 135,488 m^2^ of an experimental area for realistic disaster response exercises [29], featuring dirt roads, rubble mounds, diverse vegetation, crushed vehicles, and partially buried pipelines [30].

In this case, the high-level UGV mission specification corresponds to a casualty evacuation starting from ${\langle i,j,\theta \rangle }_{0}=\;\langle$366,991, 4,064,448, π/2〉 depicted as a red spot with an arrow for the heading. The forbidden partition, marked by black cells, represents areas occupied by victims and tents. The goal partitions, marked by yellow cells, are designated areas close enough for the UGV to access and assist the victims while maintaining a safe distance. As for the UGV slope constraints, we consider appropriate values for safe casualty evacuation, i.e., $\alpha_{\min}=-\pi /18\;\mathrm{rad}\;\left(-10{}^{\circ}\right)$ for downhill and $\alpha_{\max}=\pi /30\;\mathrm{rad}\;\left(6{}^{\circ}\right)$ for uphill.

The mission is specified by the following LTL formula:

(18) $$\begin{array}{cc} \phi_{5}\;\overset{\mathrm{def}}{=}\; & \left(\;\;\neg \;\;\pi_{f}\;\;U\;\;\;\;\pi_{\mathrm{robotic}}\;\;U\;\;\;\;\pi_{\mathrm{medical}}\;\;U\;\;\;\;\pi_{1}\right)\;\;\;\wedge \;\;\;\\ \; & \left(\;\;\neg \;\;\pi_{f}\;\;U\;\;\;\;\pi_{\mathrm{robotic}}\;\;U\;\;\;\;\pi_{\mathrm{medical}}\;\;U\;\;\;\;\pi_{2}\right)\;,\; \end{array}$$

i.e., *the UGV must eventually reach two geolocated victims to evacuate them to a medical tent and finally return to the robotic tent*.

## 5. Experimental Results

This section discusses the resulting paths, compares experimental results of the proposed method with respect to the baseline LTL path planned described in Section 4.2, and analyzes performance.

### 5.1. Path Planning Analysis

Figure 3, Figure 4, Figure 5, Figure 6 and Figure 7 illustrate the resulting paths (red lines) for the UGV example missions, Equations (14)–(18), respectively. The arrows are color-coded to show the UGV’s motion toward each goal partition in the mission sequence. The same color is used in the macron mark above the corresponding partition number in the goal cell. Different colors in the arrows indicate the UGV motion toward the next goal partition to be reached.

For the synthetic DEM, Figure 3, Figure 4, Figure 5 and Figure 6 show results for the different on-cell turn constraints Θ. In the first three missions, only Θ={0,±π/2} and Θ={0,±π/4,±π/2} produce result paths. The limited manoueverability of Θ={0,±π/4}, which does not allow π/2 turns, prevents paths from being found without encountering obstacles or reaching the DEM boundary limits. This limitation is evident in the wide loop around the elevated area on top of Figure 6b for $\phi_{4}$, which is the only mission that achieves a path with this motion constraint.

In Figure 3, both paths satisfy the goal partition order specified by $\phi_{1}$. However, different turn constraints cause partition $X_{3}$ to be reached at different states. The shortest path length (84 vs. 109 states) is achieved with the least restrictive maneuverability (i.e., Figure 3b). Figure 4 corresponds to $\phi_{2}$, which does not impose a specific order. As a result, the goal partition sequence is different: $X_{2},X_{4},X_{3},$ and $X_{1}$ for Θ={0,±π/2} (see Figure 4a), and $X_{1},X_{3},X_{4},$ and $X_{2}$ for Θ={0,±π/4,±π/2} (see Figure 4b) by reducing the path length (93 vs. 70). Figure 5 corresponds to $\phi_{3}$, where the order for the last two partitions was not specified. In this case, both turn constraints yield the same goal partition sequence, even if the least restrictive manoeuverability (see Figure 5b) produces a shorter path (80 vs. 105). Figure 6 corresponds to $\phi_{4}$, a simpler mission that only needs to reach $X_{4}$ before returning to the initial position (in any heading). This figure illustrates the difference between the path length in terms of states (seen as the number of traversed cells), which is the LTL optimization criterion imposed by SPIN, and the actual path length (since diagonal cell motions are longer). Thus, paths in Figure 6a–c have 55, 48, and 44 states that correspond to 54 m, 54.9 m, and 47.6 m, respectively.

As for the real DEM, Figure 7 illustrates the path for the multi-victim evacuation mission $\phi_{5}$ specified by Equation (18). In particular, the figure shows the path for Θ={0,±π/2}. Figure 7a reveals that the resulting path is close to the dirt roads in the environment, which correspond mostly with low terrain gradients (as seen in Figure 7b).

### 5.2. Comparison with the Baseline Path Planner

Table 2 offers a comparison of the proposed method against the baseline LTL path planner (see Section 4.2) for mission specifications in synthetic ($\phi_{1}$ to $\phi_{4}$) and real-world ($\phi_{5}$) DEMs. The table presents the total computation time $t_{\mathrm{total}}$ and partial times for the LTL workflow processes (see Figure 2): $t_{\mathrm{SPIN}}$ for the generation of the LTL verifier in C, $t_{\mathrm{comp}}$ for the compilation, $t_{\mathrm{search}}$ for the breadth-first search for the counterexample, and $t_{\mathrm{map}}$ for the mapping of the shortest path. Additionally, the table shows the number of explored states and the required memory during the counterexample search phase, as well as the length (i.e., the number of $A_{S}$ states) of the resulting shortest path. Furthermore, the path for each LTL mission specification has been computed for three different sets Θ of allowed on-cell turns (see Section 3.1).

Both LTL path planners leverage SPIN’s specific search optimization techniques, which reduce the number of explored states and the required memory. Consequently, the memory requirements and the number of explored states are the same for both methods. The state-space explosion problem (i.e., reaching the memory limit of 16,384 MB in our hardware platform) occurs for both cases with ℓ=8 in the real-world DEM, so no resulting paths are provided. This can be explained by the real-world DEM having a significantly larger number of cells and a higher ratio of children per node due to the smoother terrain compared to the synthetic DEM.

To achieve a solution with the available hardware, we selected the lossy “bitstate hashing” (-DBITSTATE) compression method in the compile options. Table 2 shows the results using both -DCOLLAPSE and -DBITSTATE. With the lossy method, the computational time for $\phi_{5}$ with Θ={0,±π/2} was reduced by 70.4%, the state-space explosion problem was mitigated (reducing the required memory by 88.2%), and the resulting suboptimal path length was equal to the shortest path length obtained with lossless compression.

All in all, Table 2 shows an average reduction of 52.4% in $t_{\mathrm{total}}$ with our proposal compared to the baseline planner. The most remarkable decrease occurs in $t_{\mathrm{SPIN}}$, where the lower complexity of the LTL property reduces the generation time of the C-file verifier (37.2%) and, consequently, the compilation time $t_{\mathrm{comp}}$ (72%).

### 5.3. Performance Analysis

The results for different DEM sizes in Table 2 (i.e., 17×17 for $\phi_{1}$ to $\phi_{4}$ in the synthetic DEM, and 292×232 for $\phi_{5}$ in the real-world DEM) indicate that DEM size mainly affects $t_{\mathrm{search}}$ but is not relevant for other processes of the SPIN workflow. The real-world environment is larger and has a smoother relief, which causes a higher number of states and valid transitions in the system model $A_{S}$. This can explain the higher memory (about 16 GB, for the real-world mission vs. 160 MB for synthetic DEMs) needed to save the nodes expanded (about $3.9\times 10^{6}$ vs. $0.26\times 10^{6}$, respectively) by the BFS algorithm.

The set Θ also influences the counterexample search time. Thus, the less restrictive set Θ={0,±π/4,±π/2} implies a higher ratio of children per node in each BFS iteration, which increases $t_{\mathrm{search}}$ and $t_{\mathrm{total}}$ but could reduce the resulting path length. For Θ={0,±π/4}, a larger number of traversable neighboring cells are required to move through the environment, which, in some cases (e.g., $\phi_{1}$, $\phi_{2}$ and $\phi_{3}$), prevents finding a path that satisfies the mission.

The complexity of the system model and/or the LTL formula describing the high-level mission specification, i.e., the number of states of the automaton product $A_{S}⊗A_{\;\;\neg \;\;\phi }$, affects $t_{\mathrm{SPIN}}$ and $t_{\mathrm{comp}}$. Thus, for LTL formulas with lower complexity, i.e., $\phi_{4}$ and $\phi_{5}$, these times are shorter. $t_{\mathrm{map}}$ is irrelevant, with values between 100 and 500 ms, increasing slightly for longer path lengths.

## 6. Conclusions

In this paper, we proposed a SPIN-based linear temporal logic (LTL) path planner for uncrewed ground vehicle (UGV) missions on uneven terrain. Our approach leverages existing search optimization techniques available in the open-source Simple Promela Interpreter (SPIN) model checker. First, the system model incorporates UGV motion constraints for a digital elevation model (DEM), including factors such as asymmetric slope limitations and maneuverability constraints like on-cell turns. These constraints are integrated into the system model, allowing them to be omitted from the LTL model checking process and focusing solely on verifying mission specifications. To enhance the efficiency of state-space exploration, we defined two general SPIN-efficient LTL formulas for specifying UGV missions. These formulas allow for reaching a set of goal DEM partitions in either a specified or unspecified order. The proposed planner was implemented using SPIN with customized search optimization parameters.

For experimental analysis, the planner was validated on both synthetic and real-world DEMs. Specifically, we tested four representative high-level missions on a synthetic DEM and applied the framework to plan a multi-victim evacuation mission in a real large-scale DEM of 135,488 m^2^. The experimental results show that our approach significantly reduces the total planning time, particularly in the generation and compilation of the LTL verifier’s source code, compared to a baseline planner. While both approaches apply SPIN’s search optimization techniques, resulting in no significant differences in path search time, our method demonstrates clear advantages in the preparatory stages of the planning process. Regarding the observed limitations, the proposed method encounters scalability challenges in large-scale DEMs. This is a common issue for graph-search approaches.

In future work, it will be valuable to explore enhancements to search algorithms in directed weighted transition systems that model UGV motion over DEMs with a broader set of motion constraints. These constraints could encompass non-uniform velocities and any-angle motion primitives. Furthermore, focusing on optimizing travel time rather than just the number of cells traversed may offer a more effective criterion for path planning in time-critical missions within challenging environments. Furthermore, future work will address the integration of the planner into the control architecture of a UGV with appropriate path tracking and motion control methods for rough terrain.

## Figures and Tables

**Table 1:** Simplified linear temporal logic formulas (LTL) for high-level uncrewed ground vehicle (UGV) missions.

F=⌀	Returns	LTL Formula ϕ
|G|=1		
no	no	$◊\pi_{1}$
yes	no	$\;\;\neg \;\;\pi_{f}\;\;U\;\;\;\;\pi_{1}$
no	yes	$◊\pi_{r}\;\;U\;\;\;\;\pi_{1}$
yes	yes	$\;\;\neg \;\;\pi_{f}\;\;U\;\;\;\;\pi_{r}\;\;U\;\;\;\;\pi_{1}$
|G|>1 and in a given order		
no	no	$◊\pi_{\left|G\right|}\;\;U\;\;\;\overset{1}{\underset{ı=|G|-1}{⋃}}\pi_{ı}$
yes	no	$\;\;\neg \;\;\pi_{f}\;\;U\;\;\;\overset{1}{\underset{ı=|G|}{⋃}}\pi_{ı}$
no	yes	$◊\pi_{r}\;\;U\;\;\;\overset{1}{\underset{ı=|G|}{⋃}}\pi_{ı}$
|G|>1 and no specific order		
no	no	$\overset{\left|G\right|}{\underset{ı=1}{⋀}}◊\pi_{ı}$
yes	no	$\overset{\left|G\right|}{\underset{ı=1}{⋀}}\left(\;\;\;\neg \;\;\pi_{f}\;\;U\;\;\;\;\pi_{ı}\;\right)$
no	yes	$\;\overset{\left|G\right|}{\underset{ı=1}{⋀}}◊\pi_{r}\;\;U\;\;\;\;\pi_{ı}$

**Table 2:** Comparison of the proposed method against the baseline LTL path planner in synthetic and real-world DEMs: computation times, explored states, required memory, and path length.

Neighborhood and Θ	$t_{\mathrm{SPIN}}$ (s)	$t_{\mathrm{comp}}$ (s)	Explored States	Memory (MB)	$t_{\mathrm{search}}$ (s)	$t_{\mathrm{map}}$ (s)	Path Length	$t_{\mathrm{total}}$ (s)
*Synthetic DEM:*	ours/base	ours/base	ours/base	ours/base	ours/base	ours/base	ours/base	ours/base
LTL formula $\phi_{1}$, Equation (14)								
ℓ=4,Θ={0,±π/2}	0.47/0.97	3.10/28.58	20,774/20,774	36/36	0.33/0.35	0.11/0.12	109/109	4.02/30.02
ℓ=8,Θ={0,±π/4}	0.46/1.02	3.11/27.05	3619/3619	36/36	0.17/0.20		no path	3.74/28.27
ℓ=8,Θ={0,±π/4,±π/2}	0.47/1.06	3.12/28.07	78,892/78,992	136/136	0.92/1.09	0.12/0.12	84/84	4.63/33.34
LTL formula $\phi_{2}$, Equation (15)								
ℓ=4,Θ={0,±π/2}	0.52/1.07	3.03/28.75	69,348/69,348	36/36	0.60/0.69	0.12/0.13	93/93	4.28/30.65
ℓ=8,Θ={0,±π/4}	0.52/1.03	3.08/27.19	7243/7243	36/36	0.19/0.25		no path	3.79/28.47
ℓ=8,Θ={0,±π/4,±π/2}	0.52/1.06	3.12/27.86	261,020/261,020	136/136	2.07/3.04	0.12/0.13	70/70	6.41/32.10
LTL formula $\phi_{3}$, Equation (16)								
ℓ=4,Θ={0,±π/2}	0.48/1.05	3.22/34.88	25,408/25,408	36/36	0.34/0.41	0.13/0.15	105/105	4.17/36.49
ℓ=8,Θ={0,±π/4}	0.49/1.17	3.34/32.95	3619/3619	36/36	0.17/0.21		no path	4.00/34.33
ℓ=8,Θ={0,±π/4,±π/2}	0.49/1.32	3.34/34.45	95,251/95,251	136/136	1.05/1.24	0.10/0.13	80/80	4.99/37.13
LTL formula $\phi_{4}$, Equation (17)								
ℓ=4,Θ={0,±π/2}	0.36/0.34	0.92/1.09	8156/8156	36/36	0.21/0.21	0.12/0.13	55/55	1.61/1.77
ℓ=8,Θ={0,±π/4}	0.33/0.37	0.95/1.12	6478/6478	36/36	0.18/0.18	0.13/0.14	48/48	1.60/1.81
ℓ=8,Θ={0,±π/4,±π/2}	0.36/0.36	0.97/1.18	31,078/31,078	136/136	0.46/0.46	0.10/0.11	44/44	1.90/2.13
*Real-world DEM:*								
LTL formula $\phi_{5}$ Equation, (18), with |-DCOLLAPSE| compile time option								
ℓ=4,Θ={0,±π/2}	0.43/0.58	2.09/8.44	1,041,104/1,041,104	>16,045/16,045	229.79/237.39	0.46/0.42	315/315	232.94/246.94
ℓ=8,Θ={0,±π/4}	0.41/0.55	2.15/8.21	state explosion	>16,384/>16,384	38.16/38.68			40.81/47.56
ℓ=8,Θ={0,±π/4,±π/2}	0.42/0.59	2.15/8.45	state explosion	>16,384/>16,384	52.30/53.14			54.95/62.24
LTL formula $\phi_{5}$, Equation (18), with |-DBITSTATE| compile time option								
ℓ=4,Θ={0,±π/2}	0.41/0.50	2.14/8.49	1,030,053/1,028,812	>0 1900/1900	66.26/67.10	0.16/0.15	315/315	68.97/76.25
ℓ=8,Θ={0,±π/4}	0.39/0.53	2.05/8.07	1,720,797/1,740,253	>0 3600/3600	106.30/113.37	0.15/0.16	245/247	108.90/122.14
ℓ=8,Θ={0,±π/4,±π/2}	0.38/0.55	2.06/8.22	3,861,489/3,885,223	>0 6800/7000	386.84/399.17	0.15/0.15	243/244	389.44/408.09

## Data Availability

The data presented in this study are available upon request from the corresponding authors.

## Abbreviations

The following abbreviations are used in this manuscript:

2D	two-dimensional;
BFS	breadth-first search;
DEM	digital elevation model;
LTL	linear temporal logic;
NuSMV	new symbolic model verifier;
Promela	process meta language;
SPIN	simple Promela interpreter;
TuLiP	temporal logic planning;
UGV	uncrewed ground vehicle;
Uppaal	Uppsala-Aalborg.
